# Integrated Alcohol Use and Sexual Assault Prevention Program for College Men Who Engage in Heavy Drinking: Randomized Pilot Study

**DOI:** 10.2196/47354

**Published:** 2023-11-23

**Authors:** Lindsay M Orchowski, Jennifer E Merrill, Daniel W Oesterle, Nancy P Barnett, Brian Borsari, Caron Zlotnick, Michelle P Haikalis, Alan D Bekowitz

**Affiliations:** 1 Rhode Island Hospital Providence, RI United States; 2 Department of Psychiatry and Human Behavior Alpert Medical School of Brown University Providence, RI United States; 3 Brown University/Lifespan Center for Digital Health Providence, RI United States; 4 Center for Alcohol and Addiction Studies Brown University Providence, RI United States; 5 Department of Psychological Sciences Purdue University West Lafayette, IN United States; 6 San Francisco Veterans Affairs Health System San Francisco, CA United States; 7 Department of Psychiatry University of California, San Francisco San Francisco, CA United States; 8 Indepedent Researcher and Practitioner Mount Shasta, CA United States

**Keywords:** sexual assault, alcohol use, prevention, student, men, alcoholism, college, intervention, program, peer engagement

## Abstract

**Background:**

Sexual assault is prevalent on college campuses and most commonly is perpetrated by men. Problematically, there is a dearth of evidence-based prevention programs targeting men as perpetrators of sexual aggression. The Sexual Assault and Alcohol Feedback and Education (SAFE) program is an integrated alcohol and sexual assault prevention intervention for college men who engage in heavy drinking that aims to address sexual aggression proclivity and alcohol use outcomes by incorporating social norms theory, bystander intervention, and motivational interviewing.

**Objective:**

This study aims to examine the initial feasibility-, acceptability-, and efficacy-related outcomes of a randomized pilot trial of an integrated alcohol and sexual assault prevention program for college men who engage in heavy drinking.

**Methods:**

This study included 115 college men who engaged in heavy drinking, who were randomly assigned to the SAFE program or a mindfulness-based control condition (MBCC). The feasibility of implementation, adequacy of participant retention, fidelity and competency of program administration, and satisfaction and utility of the intervention were evaluated. The primary outcomes of alcohol use and sexual aggression were evaluated at 2 and 6 months after baseline. The secondary outcomes of perceived peer norms, risks for sexual aggression, and bystander intervention were also assessed. The extent to which the motivational interviewing session with personalized normative feedback facilitated changes in the proximal outcomes of drinking intentions, motivation to change, and self-efficacy was also examined.

**Results:**

The study procedures resulted in high program completion and retention (>80%), high fidelity to the program manual (>80% of the content included), high competency in program administration, and high ratings of satisfaction and program utility in addressing sexual relationships and alcohol use. Both groups reported declines in the number of drinks per week and number of heavy drinking days. Compared with the MBCC participants, the SAFE participants reported higher motivation to change alcohol use after the program, as well as greater use of alcohol protective behavioral strategies at 6 months. Compared with the MBCC participants, the SAFE participants also reported lower perceived peer engagement in sexual coercion, perceived peer comfort with sexism, and peer drinking norms at 2 and 6 months. However, no group differences were observed in sexual aggression severity, rape myth acceptance, or the labeling of sexual consent. Results regarding bystander intervention intentions were mixed, with the MBCC group showing decreased intentions at 2 months and the SAFE group reporting increased intentions at both 2 and 6 months.

**Conclusions:**

The findings provide promising evidence for the feasibility, acceptability, utility, and preliminary efficacy of the SAFE program in reducing alcohol use and positively influencing perceived peer norms and intentions for bystander intervention among college men who drink.

**Trial Registration:**

ClinicalTrials.gov NCT05773027; https://clinicaltrials.gov/study/NCT05773027

## Introduction

### Background

Although anyone can experience or perpetrate sexual violence, most acts of sexual violence are perpetrated by men against women [1]. According to a seminal study of sexual violence on college campuses by Koss and her colleagues [2], 25.1% of college men reported perpetrating some form of sexual aggression (ie, perpetrated rape, attempted rape, sexual coercion, and unwanted sexual contact) since the age of 14 years. Over 30 years later, college men continue to report alarmingly high rates of sexual aggression [3]. Research has found that 19.5% of college men report engaging in some form of sexual aggression over the course of a semester [4], and 34.5% of college men report engaging in some form of sexual aggression over a 4-year period [5]. Most sexual assaults on college campuses occur between individuals who know each other [6]. Further, approximately half of all assaults involve alcohol use by the victim, the perpetrator, or both parties [7,8]. Alcohol use at the time of an assault is associated with increased aggression [9], increased assault severity [10], and a greater likelihood of victim injury [11,12].

Although not all men who perpetrate sexual aggression do so under the influence of alcohol [13,14], data document higher rates of alcohol use and heavy drinking among college men who perpetrate sexual aggression than among those with no such history [15,16]. College men who engage in heavy drinking also report visiting high-risk drinking environments with the intention of locating a sexual partner, who they assume will be open to a sexual advance [17]. Alcohol can influence the risk for sexual aggression in several ways [7]. For example, the pharmacological effects of alcohol may draw men’s attention to the most salient cues in their environment [18], thereby increasing the likelihood of misinterpreting sexual interest [19]. Impairments in impulse control [20] and reductions in tension and anxiety [21,22] when drinking may also increase the risk for sexual aggression. Expecting to feel sexual or aggressive when drinking [23], men may also consume alcohol with the intention of committing acts of sexual aggression [24,25]. Taken together, these findings suggest that men who engage in heavy drinking may be particularly important to target in sexual assault prevention efforts.

In addition to alcohol use, numerous salient individual risk factors have been established as increasing men’s likelihood of perpetrating sexual aggression, including attitudinal risk factors, as well as beliefs and expectations men hold surrounding sexual communication and consent. The effect of rape myth acceptance on sexual aggression is particularly well established [26]. More specifically, rape myth acceptance refers to a set of stereotyped beliefs that blame victims, condone sexual violence, and minimize perpetrator responsibility [27]. Hypergender ideological beliefs also confer a significant risk of perpetrating sexual aggression [28]. Unlike rape myth acceptance beliefs, which almost exclusively refer to rape- and sexual assault–specific attitudes, hypergender ideological beliefs capture a range of broad stereotypical attitudes and expectations surrounding gender roles. Specifically, hypergender ideological beliefs refer to both hypermasculine (ie, it is okay for a man to be a little forceful to get sex) and hyperfeminine (women instinctively try to manipulate men) roles that men expect other men and women to adhere to. It is also important to acknowledge the complex role of how college men who engage in heavy drinking conceptualize consent as another important factor that contributes to men’s sexual aggression [17].

Despite the prevalence of sexual aggression among college men, a limited number of prevention programs demonstrate reductions in the rate of sexual aggression among college men [29]. Only 2 sexual assault prevention programs designed for college men show promise for producing short-term changes in attitudes, behaviors, and the perpetration of sexual aggression among college men [30,31]. In an evaluation of the Men’s Workshop, Gidycz and colleagues [30] found that men living in first-year residence halls who participated in the 2.5-hour prevention program grounded in social norms theory and bystander intervention skills reported lower rates of sexual aggression over a 4-month follow-up than men in a wait-list control group. Real Consent [31] is another sexual assault prevention program for college men, which consists of six 30-minute web-based modules. Men who participated in the program reported lower rates of sexual violence perpetration and increased intentions to engage in bystander intervention to prevent sexual violence over a 6-month follow-up in comparison with men in the control group [31]. Although neither program evaluation examined whether the program was effective in reducing perpetration risk among men with heavy drinking, it is possible that the interventions may be more salient if tailored to address perpetration proclivity among this high-risk group.

A limited number of integrated alcohol and sexual assault prevention approaches exist for college men who engage in heavy drinking. Integrated alcohol and sexual assault prevention programs operate under the premise that prevention efforts for these intersecting problems can be more effective if men are provided with evidence-based alcohol interventions to reduce drinking as well as theory-driven sexual assault prevention efforts to change the attitudes and behaviors that drive sexual aggression [32]. Gilmore et al [33] conducted a study of 24 undergraduate students to document the usability and preliminary outcomes of a web-based alcohol and sexual assault prevention program for college students that included tailored content for men to address sexual aggression. Orchowski et al [34] also conducted a small open pilot trial to evaluate a 3-session program specifically designed for college men who engage in heavy drinking, which integrates active alcohol use intervention and sexual assault prevention. Specifically, the *Sexual Assault and Alcohol Feedback and Education (SAFE)* program begins with a 90-minute individually administered review of personalized normative feedback (PNF) addressing alcohol use, sexual activity, alcohol-related sexual consequences, the understanding of consent, and engagement in bystander intervention. PNF involves providing participants with information about the behaviors or attitudes of similar peers (eg, same age, cohort, or gender) and contrasting this information with the trainee’s own behaviors or perceptions. This session is informed by intervention techniques used in Brief Alcohol Screening and Intervention for College Students [35] as well as motivational interviewing (MI) [36]. MI is a nonconfrontational approach that assists individuals in increasing their motivation to change a given target behavior through the resolution of ambivalence. The SAFE intervention is also grounded in social norms theory [37] and bystander intervention skills training [38]. Social norms theory proposes that men’s engagement in sexual aggression is in part driven by misperceptions regarding the acceptability and prevalence of sexual aggression among other men and works to change the proclivity for violence by providing men with information to correct these misperceptions [39]. Bystander intervention is an approach to violence prevention that encourages all members of a community to respond prosocially to stop or interrupt markers of risk for sexual assault. On the basis of these theories, in SAFE, men complete a 2.5-hour group-based sexual assault prevention workshop focusing on social norms, empathy, masculinity, consent, and bystander intervention. The session is informed by the content of the Men’s Workshop [30,39] and includes additional content addressing the way in which alcohol use influences sexual communication, consent, and bystander intervention. Men also complete a 90-minute booster group session review of program material 2 months following program participation focused on how they applied the information and skills learned in the program over the interim. Orchowski and colleagues [34] found that the intervention was feasible and acceptable among the participants. Given that SAFE is currently the only integrated alcohol and sexual assault prevention program designed for in-person administration to college men who engage in heavy drinking, comparison with a control condition is warranted to better establish the feasibility and acceptability of the program and research procedures and to examine program outcomes.

### Purpose of This Study

The purpose of this study was to conduct a pilot randomized evaluation of the SAFE program to assess the preliminary feasibility and acceptance of the program among college men who engage in heavy drinking and to examine preliminary program outcomes in comparison with an active attention- and dose-matched control group (NCT05773027). A mindfulness-based control condition (MBCC) with the same number of sessions, duration, modality (ie, in-person), and administration format (ie, individually administered PNF session, a group-based workshop, with a group-based booster session) served as the control condition. Mindfulness-based intervention for alcohol use is a growing field of study [40-42]. MBCC was chosen as an active comparison condition, given prior studies documenting the positive impacts of relaxation training on alcohol use among young adults [43,44]. Emotion regulation, which is a target of mindfulness, is increasingly recognized as a mechanism of sexual aggression [45,46]. In fact, prior research found that drinking was positively associated with sexual aggression among men with low levels of mindfulness [47].

The overall objective of this study was to evaluate the feasibility, acceptability, and preliminary outcomes of the SAFE program. The aims and methodology of this study align with those of a stage 1B intervention development study [48-50]. As described by Rounsaville et al [50], the aims of a stage 1B study are to gauge the acceptance of the new intervention, document the research team’s ability to recruit and retain the sample, and establish the feasibility of intervention delivery in a particular setting.

### Primary Research Questions

The primary research questions in Textbox 1 were explored.

**Research questions.**

**Research question 1**: **feasibility of intervention delivery**

- Are the Sexual Assault and Alcohol Feedback and Education (SAFE) and mindfulness-based control condition interventions feasible to implement, as evidenced by >80% of the participants completing all sessions?

**Research question 2: long-term retention rates**

- Are the retention procedures sufficient to ensure adequate completion of follow-up surveys over time, as evidenced by >80% of the participants completing the 2- and 6-mo follow-up surveys?

**Research question 3: adherence**

- Was SAFE administered with fidelity to the protocol, as evidenced by >80% of the program components being administered according to the manual?

**Research question 4: competency**

- Did the facilitators of SAFE demonstrate high levels of competency in motivational interviewing skills, as demonstrated by high external ratings (ie, Motivational Interviewing Treatment Integrity global ratings ≥4) of session 1 of the intervention, and through high participant ratings of the use of a nonjudgmental style (ie, average scores >3 on a scale ranging from 1 to 4)?

**Research question 5**: **satisfaction and utility**

- Does the SAFE program demonstrate high levels of participant satisfaction and perceived program utility?

### Secondary Research Questions

Although pilot studies have been used to assess changes in participant outcomes over time, document effect sizes, and determine the sample size needed for a subsequent trial [50], there is recognition that the effect sizes that result from small pilot studies are unstable and result in a high level of type II error [51-53]. As such, outcome analyses are often included in pilot randomized trials to gauge the preliminary direction of effects and examine trends rather than to document effect sizes [51]. Accordingly, a series of exploratory analyses was conducted to examine the extent to which participants in the SAFE group reported changes in the primary behavioral outcomes, as well as putative mechanisms of program effects, when compared with participants in the mindfulness-based control group that received relaxation and mindfulness skills training.

Specifically, at the 2- and 6-month follow-up assessments, we examined whether SAFE varied from MBCC in the primary target outcomes of alcohol use and sexual aggression. At the 2- and 6-month follow-up assessments, we also examined whether SAFE varied from MBCC in the secondary proximal outcomes of peer norms (ie, alcohol use, comfort with sexism, engagement in sexual coercion, and bystander intervention), risk factors for sexual aggression (ie, rape myth acceptance, hypergender ideology, and the labeling of consent), and bystander intervention intentions and confidence. As putative mechanisms of the effects of the MI session with PNF (BMI+PNF; session 1), we also examined whether the SAFE participants reported in motivation and confidence to reduce their alcohol use as well as drinking intentions from before the intervention to after the intervention.

## Methods

### Participants and Study Eligibility Criteria

Participants included 115 men aged between 18 and 22 years currently enrolled at a large northeastern public university in the United States. The demographic characteristics of the participants are reported in Table 1. Inclusion criteria for the study included engaging in 2 or more episodes of binge drinking in the past month, defined by the Substance Abuse and Mental Health Services Administration [54] as the consumption of ≥5 drinks per day for men on the same occasion. Given that the sexual assault prevention program focused on men’s engagement in sexual aggression against women, the inclusion criteria also included engaging in oral, vaginal, or anal sexual intercourse with a female partner ≥1 times in the past 4 months. Sexual activity in the past 4 months was included as a part of the study eligibility criteria to garner a sample that might be at the highest risk for perpetrating sexual aggression against a female partner. Exclusion criteria included displaying symptoms consistent with alcohol use withdrawal, reporting current suicidal or homicidal ideation, or meeting criteria consistent with a diagnosis of antisocial personality disorder (ASPD). Participants meeting the diagnostic criteria for ASPD were excluded because ASPD, often referred to as sociopathy, is a personality disorder that is often resistant to treatment. As such, it was believed that participants meeting the criteria for ASPD would be unlikely to benefit from a multisession preventive intervention. Table 1 summarizes the characteristics of the participants in total and by intervention group.

**Table 1:** Participant characteristics.

Characteristics		Total sample (N=115)	SAFE^a^ (n=58)	MBCC^b^ (n=57)
Age (years), mean (SD)		19.98 (1.41)	19.88 (1.34)	20.09 (1.49)
**Year in school, n (%)**				
	Freshman	22 (19.1)	8 (13.8)	14 (24.6)
	Sophomore	30 (26.1)	17 (29.3)	13 (22.8)
	Junior	33 (28.7)	20 (34.5)	13 (22.8)
	Senior	28 (24.3)	12 (20.7)	16 (28.1)
	Unknown	2 (1.7)	1 (1.7)	1 (1.8)
**Race, n (%)**				
	Asian	2 (1.7)	2 (3.4)	0 (0)
	Black	4 (3.5)	2 (3.4)	2 (3.5)
	White	101 (87.8)	52 (89.7)	49 (86)
	Other or unknown	8 (7)	2 (3.4)	6 (10.5)
**Ethnicity, n (%)**				
	Hispanic or Latino	9 (7.8)	1 (1.7)	8 (14)
	Non-Hispanic Latino	102 (88.7)	54 (93.1)	48 (84.2)
	Not reported	4 (3.5)	3 (5.2)	1 (1.8)
**Dating status, n (%)**				
	Dating casually	57 (49.6)	28 (48.3)	29 (50.9)
	Not dating	21 (18.3)	10 (17.2)	11 (19.3)
	Long-term relationship	37 (32.2)	20 (34.5)	17 (29.8)
**Social fraternity, n (%)**				
	Yes	37 (32.2)	18 (31)	19 (33.3)
	No	78 (67.8)	40 (69)	38 (66.7)
**Athletic team, n (%)**				
	Yes	13 (11.3)	7 (12.1)	6 (10.5)
	No	97 (84.3)	49 (84.5)	48 (84.2)
	No, but was previously	5 (4.3)	2 (3.4)	3 (5.3)

^a^SAFE: Sexual Assault and Alcohol Feedback and Education.

^b^MBCC: mindfulness-based control condition.

### Recruitment and Screening Procedures

Men were recruited for the study using a list from the university registrar of all men currently enrolled at the university aged between 18 and 22 years. Potential participants were included in this list if they were identified as a “man” by the university registrar. A random sample of 10,500 students received an email with a link to a confidential web-based study screening portal with a secure https connection, 128-bit encryption, and a signed Secure Sockets Layer certificate. The study was described as addressing alcohol consumption and dating among college men. After entering the screening portal, the participants were presented with an electronic consent statement. Men who agreed to the electronic consent statement were entered into the self-administered web-based screening portal. Past month alcohol use was assessed using the graduated frequency measure [55], and a single item assessed the number of female sexual partners in the past 4 months. Among every 50 participants who completed the screening, 1 was randomly selected to receive a US $50 gift card. After screening, men were directed to a separate questionnaire to enter their email address to be contacted regarding their eligibility for the larger study.

**Figure 1:**
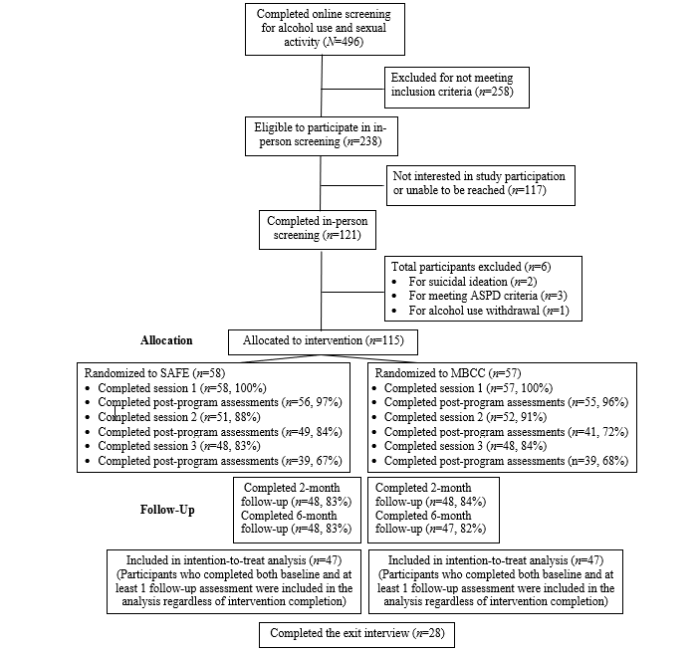
Participant recruitment and retention. ASPD: antisocial personality disorder. MBCC: mindfulness-based control condition. SAFE: Sexual Assault and Alcohol Feedback and Education.

A total of 496 men completed the web-based screening, of whom 238 (48%) met the alcohol use and sexual activity inclusion criteria. Men who met the inclusion criteria were provided with a description of the larger study via phone and invited to an in-person screening and baseline assessment. Of these 238 individuals, 121 (50.8%) presented for the in-person screening (Figure 1). The in-person screening was administered by a trained male research assistant. Specifically, the research assistant administered the Alcohol Use Withdrawal Symptom Checklist to the participants [56]. Men were excluded from the study if they scored ≥23 on the Alcohol Use Withdrawal Symptom Checklist. A single question from the Beck Depression Inventory [57] assessed current suicide risk, and a single question assessed current homicidal ideation. The ASPD module of the Structured Interview for Diagnostic and Statistical Manual of Mental Disorders, Fourth Edition Personality [58] assessed characteristics consistent with ASPD. Men were excluded if they engaged in ≥3 domains of problem behavior and expressed no remorse for their actions. A total of 6 men were excluded from the study. All 115 men who were determined to be eligible for the study decided to enroll, were randomized to a condition, provided informed consent, and completed the baseline assessment.

### Ethical Considerations

The study procedures were approved (project number 413813) by the first author’s hospital institutional review board and by the local university where the participants were recruited and completed the intervention. A certificate of confidentiality was received from the National Institutes of Health. Assessment and intervention sessions were completed in private individual or group interview rooms within the psychology training clinic at a local university. Informed consent was obtained in person during session 1. The participants were informed that their study data would be deidentified.

Session 1 was completed in 3.5 hours, including the in-person screening, informed consent procedures, baseline assessment, session 1 of SAFE or MBCC, and postsession satisfaction surveys. Men were compensated with US $40. Session 2 was offered 2 weeks following session 1. An orientation to the session (eg, confidentiality considerations), group workshop, and postprogram surveys were completed within 3 hours. Men were compensated with US $45. Men completed the 2-month assessment via a web-based survey and were compensated with US $50 before attending session 3, which was conducted 2 months after session 1. The informed consent procedures, booster session, and postprogram surveys were completed within 2 hours. Men completed follow-up assessments via a web-based survey at 6 months and were compensated US $55 for their time. Men who completed all study components received a US $30 completion bonus. Approximately 50% of the men who completed the SAFE program were randomly selected and invited to return for a 60-minute exit interview, which was completed after the 6-month follow-up assessment. The exit interview was facilitated by a male research assistant and consisted of a pencil and paper survey as well as a semistructured interview. Participants received US $30 for the interview.

### Study Procedures

The 30-minute baseline survey was completed on a laptop computer immediately after completing the in-person screening and providing study consent. Some measures were administered by a trained male interviewer, and other measures were self-administered. During a short break, the facilitator printed the PNF for session 1 of the SAFE program or prepared materials for MBCC. The PNF was programmed to be automatically generated using the DatStat Illume survey software. After session 1, participants in both intervention groups completed a series of pencil and paper questionnaires assessing program satisfaction, perceived utility of the program content, and alliance with the facilitator. Participants returned the forms to the facilitator in a sealed envelope and were provided with handouts and resources. Participants in the SAFE condition received a copy of their PNF.

### Intervention

#### Facilitators

All the sessions were conducted by a trained male program facilitator. One facilitator administered session 1, and sessions 2 and 3 were administered by a 2-person team. Five facilitators were trained to facilitate the program. Training included modules in MI, mindfulness intervention, social norms theory, bystander intervention, and risk factors for sexual aggression. Each session was also demonstrated to the facilitators. Facilitators received intensive training on how to address resistance and encourage the active engagement of participants using a nonjudgmental approach. Facilitators completed at least 2 full practice programs until they were rated as competent in program delivery, using the adherence and competency rating systems described subsequently. The program facilitators included 5 young adult men, who were postbaccalaureate research assistants or graduate students in clinical psychology doctoral programs.

#### The SAFE Program

The SAFE intervention is a multisession program that uses MI [36] and the social norms approach [37,39,59]. Session 1 of SAFE is conceptualized as a brief motivational intervention, where participants meet with a study therapist for 90 minutes, who provides PNF [35,36], and explores the participants’ alcohol use and sexual behaviors in a nonconfrontational manner to highlight discrepancies between actual and ideal behaviors [60]. Sessions 2 and 3 of SAFE use an adapted version of the Men’s Workshop [39], which is firmly rooted in the social norms approach [37,39,59]. Specifically, session 2 of SAFE is a 2-hour group-based workshop, which discusses the frequency of alcohol use, sexual activity, and sexual assault on college campuses; sexual consent; false accusations of sexual assault; and bystander intervention, and data are provided to dispel commonly held misperceptions related to sexual assault. Session 3 of SAFE is considered a “booster session” building upon the content presented in session 2. During this session, participants meet as a group for 90 minutes to continue their discussion related to sexual assault on campus and engage in small group practice to increase their competency in bystander intervention. All sessions are interactive and discussion oriented. The SAFE program was previously evaluated in an open pilot trial [34].

#### MBCC Group

To parallel the SAFE group, participants in the comparison group received a multisession mindfulness-based stress reduction program, based on prior research on mindfulness intervention [61]. The MBCC was developed for the purpose of this study. In session 1 of the control intervention, participants met one on one with a study facilitator to explore their life in college, identify lifestyle factors contributing to stress, look into the role of exercise and nutrition in their daily life and were introduced to the practice of mindfulness and meditation. Session 2 of the control intervention was a 2-hour group-based discussion to address common stressors on campus, engage in several mindfulness and meditation exercises, and learn how to apply these skills in everyday life. In session 3 of the control intervention, participants met as a group for 90 minutes to continue to build upon their mindfulness and meditation skills and discuss the ways in which mindfulness and meditation have impacted their lives since session 2.

### Measures

#### Participant Characteristics

Demographic characteristics, including age, grade, race, ethnicity, dating status, membership in a social fraternity, and participation in an athletic team, were assessed at baseline.

#### Research Question 1: Feasibility

Feasibility of the program and research protocol was assessed via program attendance rates.

#### Research Question 2: Retention

Rates of study retention were calculated at each follow-up period.

#### Research Question 3: Adherence

An outside rater assessed the fidelity to the program protocol using adherence checklists corresponding to the content of the SAFE and MBCC programs. The rater was an undergraduate volunteer who was not involved in other aspects of the study and received training by the study director. For SAFE, 34 items were assessed for session 1, 64 items were assessed for session 2, and 14 items were assessed for session 3. For MBCC, 13 items were assessed for session 1, 16 items were assessed for session 2, and 11 items were assessed rated for session 3. All the sessions were recorded and rated. Adherence to ≥80% of the content was considered acceptable.

#### Research Question 4: Competency

The facilitators’ competency in the administration of the SAFE program was first assessed via external ratings. A total of 12 administrations of session 1 in the SAFE program (representing 20% of the sessions; 5 were double coded) were randomly selected to examine competency in using the spirit of MI. The global rating scale of Motivational Interviewing Treatment Integrity (version 4.2) [62] was used to examine facilitator competency in MI style in session 1 of the SAFE program. The global rating scale consists of 4 behaviorally anchored domains (ie, cultivating change talk, softening sustain talk, empathy, and partnership), which are rated on a 5-point scale (1=“low”; 5=“high”), and records the frequency of 10 therapist behaviors (eg, questions, simple reflections, and affirmations). The participants also rated the extent to which the facilitator, for both SAFE and MBCC, used a nonjudgmental style across 9 items on a 4-point scale (1=“strongly disagree”; 4=“strongly agree”) [63]. Cronbach α ranged from .74 to .78 across sessions 1, 2, and 3. Ratings were completed for both SAFE and MBCC following the completion of each session.

#### Research Question 5: Satisfaction and Acceptability

Participants completed the Client Satisfaction Questionnaire-8 (CSQ-8) [64] after sessions 1, 2, and 3 of each SAFE intervention module. The CSQ-8 includes 8 items that are rated on a 4-point scale. Cronbach α ranged from .80 to .88 for sessions 1, 2, and 3. Following each session, the perceived utility of the program content was assessed using 10 items adapted from the study by Magill et al [63], which were rated on a scale from 1 to 3 (1=“not useful,” 2=“useful,” and 3=“very useful”). Men who completed the exit interview also completed 6 self-report survey questions to assess their satisfaction with the SAFE program (eg, the number of sessions, facilitators, likelihood of participating in a program like SAFE in the future, and extent to which the program met their needs).

#### Primary Outcomes: Alcohol Use and Sexual Aggression

Alcohol use was assessed at each time point. An interviewer administered the Timeline Follow Back measure [65] to assess the number of standard drinks per day over the past 4 weeks. On the basis of the responses to the Timeline Follow Back, the average number of drinks per week and the number of heavy drinking days in the past month (≥5 drinks for men) were derived. Protective behavioral strategy (PBS) use was assessed using the 37-item Self-Control Questionnaire [66]. Responses were provided over the past 6 weeks at baseline and over each follow-up period. Responses were provided on a scale of 1 to 5 (1=“never”; 5=“always”). Cronbach α was .91 at baseline. Engagement in sexual aggression was assessed at baseline (since the age of 14) and each follow-up via the Sexual Experiences Survey-Short Form Perpetration [67]. The participants indicated whether they used 1 of 5 tactics to engage in unwanted sex (ie, verbal pressure, criticism, taking advantage of someone too drunk to stop it, threats of harm, and force) or to attempt or complete 7 different unwanted sexual acts, ranging from unwanted contact to penetration. Scores were calculated to reflect the frequency and severity of perpetration and ranged from 0 to 63 [68].

#### Secondary Outcomes: Peer Norms, Risks for Sexual Aggression, and Bystander Intervention

Norms were assessed for alcohol use, perceived peer engagement in sexual coercion, perceived peer comfort with sexism, and perceived peer engagement in bystander intervention. Peer norms were conceptualized as perceptions of the attitudes and behaviors of other college men. The Drinking Norms Rating Form [69] assessed the perception of peer drinking norms. The participants estimated the alcohol consumption of typical same age and gender peers on each day of the week. The responses were summed to create an indicator of the perceived norm for other men’s weekly alcohol consumption. Cronbach α was .82. Perception of peer engagement in sexual coercion and perception of peer comfort with sexism were assessed using subscales from the Sexual Social Norms Inventory [70]. Higher scores reflected the belief that peers are engaging in greater levels of sexual coercion or are more comfortable with sexist language or behavior. Cronbach α values for perceived peer engagement in sexual coercion and perceived peer comfort with sexism were .88 and .79, respectively. Perceptions of peer engagement in bystander intervention were assessed using a 20-item scale [71]. Respondents indicate how likely their friends would be to engage in a range of bystander intervention behaviors, such as “ask a stranger if they need to be walked home from a party or get their friends to do so.” Items are rated from 1 to 5 (1=not at all likely; 5=very much likely) and summed for a total score. Cronbach α was .79.

Three domains of risk for sexual aggression were assessed, namely rape myth endorsement, hypergender ideology, and labeling of consent. The endorsement of rape myths was assessed using the short form of the Illinois Rape Myth Acceptance Scale [72]. Each of the 20 items were rated from 1 to 7 (1=not at all agree; 7=very much agree). Cronbach α was .81. Adherence to traditional beliefs about masculinity was assessed using the 19-item short form of the Hypergender Ideology Scale [73]. Items were rated from 1 to 6 (1=strongly disagree; 6=strongly agree). Cronbach α was .88. Men’s labeling of consensual sexual activity was assessed using a scenario depicting the perpetration of sexual aggression [74]. The participants indicated the extent to which the scenario would be considered consensual sex (1=consensual sex; 10=rape).

Three domains of bystander intervention were assessed, namely bystander intervention intentions, confidence in intervening with friends, and confidence in intervening with strangers. The 51-item Bystander Attitudes Scale assessed the likelihood of intervening in a risky situation [38]. Items were rated from 1 to 5 (1=not at all likely; 5=extremely likely) and summed for a total score. Cronbach α was .93. The 10-item Brief Intent to Help Friends and 8-item Intent to Help Strangers scales assessed confidence in engaging in bystander intervention [71]. The participants rated their confidence in performing each task from 0 to 100 (0=definitely cannot do; 100=very certain can do). The items were summed to create a total score. Cronbach α values were .81 and .92, respectively.

#### Proximal Outcomes of Session 1

Motivation to change, self-efficacy for reducing drinking, and drinking intentions were measured at baseline and immediately following session 1. The Contemplation Ladder [75] is a single-item assessment of the motivation to change drinking, with responses ranging from 0 (“no thought of drinking less”) to 10 (“taking action to drink less”). The Brief Situational Confidence Questionnaire [76] is a single-item assessment of an individual’s confidence in resisting drinking heavily in the future, with responses ranging from 0% (“not at all confident”) to 100% (“completely confident”). A weekly calendar was used to assess drinking intentions [77]. The participants were instructed to think about what their drinking pattern would be like over the next week and then asked to enter the average number of drinks they planned to consume each day of the week. The responses were summed to represent the participants’ estimation of the total number of drinks they intended to consume in the next week.

### Data Analysis Plan

Chi-square and 2-tailed *t* test analyses examined whether the participants were effectively randomized to a condition to avoid baseline differences in core outcomes between the groups. Analyses suggested that Hispanic ethnicity was the only demographic characteristic that varied as a function of group (χ^2^_1_=6.8; *P*=.03). Two-tailed *t* tests did not reveal any group differences in program outcomes at baseline. The groups did not differ at baseline in their history of sexual aggression or drinking outcomes. Summary statistics were calculated to characterize the study sample and answer research questions 1 to 5. To explore the proximal effects of session 1, 3 repeated measures analyses of variance examined the changes in motivation to change drinking, confidence in resisting drinking heavily in the future, and weekly drinking intentions between the groups from before the session to immediately after the session.

For exploratory analyses of group differences in primary and secondary outcomes, the hierarchical linear modeling (HLM; version 7.01; Scientific Software International Inc) program [78] was used to conduct HLM. HLM was appropriate, as our data were nested within participants across time and given our interest in both between-person (level 2; condition) effects and within-person effects of time (level 1) on the outcome variables. HLM analyses began with a screen for missing data. One participant (in SAFE) was listwise deleted because of failure to provide a report of sexual aggression at baseline, given our interest in this variable as an outcome. The person-period data set for full sample analyses was represented by 342 possible observations (n=114 participants × 3 assessments). Across these participants, data were missing owing to failure to complete surveys 40 (11.7%) out of 342 assessments. The distributions of all outcomes were examined and found to be normal for all drinking variables. For sexual aggression, outliers falling 3 SDs above the mean were recoded as the highest nonoutlying value plus 1 [79]. All outcome variables were standardized such that coefficients can be interpreted as effect sizes. In the HLM models, 2 time components (time 2 mo coded 0, 1, and 0; time 6 mo coded 0, 0, and 1) were added at level 1 to represent changes from baseline to 2-month and 6-month follow-ups, respectively. Condition was added at level 2 as a predictor of the intercept (ie, effect of group on the outcome at baseline) and both time effects (ie, effect of group on the outcome at 2-mo and 6-mo follow-ups). In reporting the model results, we relied on robust SEs. All intercept effects were specified as random to allow for individual variations in baseline levels and changes over time in the outcomes. Random slopes of time components were tested and retained in the majority of models (all but the model predicting the labeling of sexual consent, where the random slope was nonsignificant). Given the preliminary nature of the study and the small sample size, the results were considered significant at *P*<.05 and marginally significant at *P*<.10.

## Results

### Research Question 1: Feasibility

Program completion rates are reported in Figure 1. In SAFE and MBCC, 100% of the men who consented to participate in the study completed session 1. No participants withdrew from a session during its administration or left a session in distress. Among the 58 SAFE participants who completed the baseline assessment and session 1 (BMI+PNF), 51 (88%) returned to complete session 2 (sexual assault prevention workshop), and 48 (83%) returned to complete session 3 (booster session). Similar return rates were observed among the 57 MBCC participants, with 52 (91%) returning to complete session 2 (wellness workshop) and 48 (84%) returning to complete session 3 (booster session).

### Research Question 2: Retention

The research procedures were successful in achieving ≥80% retention at each follow-up assessment without evidence of differential dropout between the groups. Among the 58 SAFE participants, 48 (83%) returned for the 2-month follow-up survey and 48 (83%) returned for the 6-month follow-up survey. Among the 57 MBCC participants, 48 returned for the 2-month follow-up survey and 47 (82%) returned for the 6-month follow-up survey.

### Research Question 3: Adherence

Each administration of sessions 1, 2, and 3 of SAFE and MBCC was rated as adherent to the protocol (≥80% of the content included), with an average of 89.4%, 87.3% and 99.3% of the content administered in sessions 1 (an average of 30.4, SD 2.4 out of 34 items administered), 2 (an average of 55.9, SD 5.5 of 64 items administered), and 3 (an average of 13.9, SD 0.81 out of 14 items administered), respectively. A review of the audio recordings of MBCC also suggested that the sessions were administered according to the protocol (≥80% of the content included), with an average of 96.2%, 97.5%, and 84.5% of the content administered in sessions 1 (an average of 12.5, SD 1.1 out of 13 items administered), 2 (an average of 15.6, SD 1.1 out of 16 items administered), and 3 (an average of 9.3, SD 1.3 out of 11 items administered), respectively.

### Research Question 4: Competency

On the basis of the Motivational Interviewing Treatment Integrity (version 4.2) [62], ratings on global scores for session 1 suggested that the facilitators demonstrated competency in the spirit of MI (average rating on global measures ≥3). The most common therapist behaviors were providing information (mean 8.9; range 6-15, intraclass correlation [ICC]=0.97), asking questions (mean 22.8; range 11-33; ICC=0.93), and simple reflections (mean 5.8; range 2-11; ICC=0.83). The sessions did not contain any MI-inconsistent behaviors such as confrontation. Participants’ mean ratings of facilitator competency at sessions 1, 2, and 3 were also high, with average scores >3 on a scale of 1 to 4 for all the items assessed (Table 2).

**Table 2:** Participant rating of facilitator competency^a^.

Item and group		Session 1			Session 2			Session 3	
		Values, n^b^	Values, mean (SD)	Values, n		Values, mean (SD)	Values, n		Values, mean (SD)
**The facilitator(s) was/were easy to talk to**									
	SAFE^c^	56	3.91 (0.29)	49		3.92 (0.28)	38		3.87 (0.34)
	MBCC^d^	55	3.93 (0.26)	40		3.93 (0.27)	39		3.97 (0.16)
**The facilitator(s) was/were concerned about me**									
	SAFE	56	3.25 (0.86)	49		3.16 (0.80)	38		3.34 (0.75)
	MBCC	54	3.26 (0.65)	40		3.40 (0.59)	39		3.33 (0.77)
**The facilitator(s) understood me**									
	SAFE	56	3.79 (0.46)	49		3.63 (0.60)	38		3.76 (0.43)
	MBCC	55	3.86 (0.36)	40		3.70 (0.46)	39		3.72 (0.46)
**The facilitator(s) asked my ideas before presenting his own**									
	SAFE	56	3.84 (0.37)	49		3.69 (0.58)	38		3.84 (0.37)
	MBCC	55	3.91 (0.29)	40		3.93 (0.27)	39		3.74 (0.59)
**The facilitator(s) helped me talk about my own reasons for change**									
	SAFE	56	3.64 (0.59)	48		3.23 (0.78)	38		3.71 (0.52)
	MBCC	54	3.74 (0.44)	40		3.56 (0.68)	39		3.42 (0.83)
**The facilitator(s) treated me like an equal**									
	SAFE	56	3.93 (0.26)	49		3.92 (0.34)	38		3.97 (0.16)
	MBCC	55	3.96 (0.19)	40		3.95 (0.22)	39		4.00 (0.00)
**The facilitator(s) respected my ideas about how change can occur**									
	SAFE	56	3.79 (0.56)	49		3.78 (0.42)	38		3.84 (0.44)
	MBCC	54	3.93 (0.19)	40		3.85 (0.36)	39		3.95 (0.22)
**The facilitator(s) did not push me into something I was not ready for**									
	SAFE	56	3.91 (0.29)	49		3.80 (0.50)	38		3.84 (0.37)
	MBCC	55	3.95 (0.23)	40		3.95 (0.22)	39		3.90 (0.31)
**The facilitator(s) accepted that I might choose not to change**									
	SAFE	56	3.79 (0.49)	48		3.65 (0.56)	38		3.71 (0.57)
	MBCC	55	3.92 (0.26)	40		3.88 (0.33)	39		3.77 (0.43)
**Mean score**									
	SAFE	56	3.76 (0.28)	49		3.64 (0.32)	38		3.77 (0.31)
	MBCC	55	3.83 (0.20)	40		3.79 (0.25)	39		3.76 (0.27)

^a^Item responses range from 1 to 4 (1=strongly disagree; 4=strongly agree).

^b^Reflects the number of participants who completed the item or scale.

^c^SAFE: Sexual Assault and Alcohol Feedback and Education.

^d^MBCC: mindfulness-based control condition.

### Research Question 5: Satisfaction With and Utility of the SAFE Program

The mean satisfaction ratings on the CSQ-8 among the SAFE participants completing session 1, session 2, and session 3, respectively, were 3.52 (SD 0.36), 3.54 (SD 0.42), and 3.66 (SD 0.44) on the 4-point scale. Participant ratings of the 6 major components of session 1 reflected high average ratings of program utility (ie, “useful” to “very useful”), with ratings ranging from 2.16 (SD 0.80) to 2.57 (SD 0.50) on the 3-point scale. The perceived utility of the 3 major components of sessions 2 and 3 was also high, with average ratings ranging from 2.21 (SD 1.02) to 2.57 (SD 0.87; Table 3). Of the 28 men who were selected to complete an exit interview after completing SAFE, 27 (96%) indicated that they were “moderately satisfied” or “very satisfied” with the program, number of sessions, and facilitators. Most participants indicated that they would “definitely” seek a program like this for sexual relationships. Most participants reported that they would “definitely” seek a program like this in the future to address alcohol use. All participants indicated that most or all of their needs were met by the program (Table 4).

**Table 3:** Utility of the Sexual Assault and Alcohol Feedback and Education program content^a^.

Item	Session 1			Session 2			Session 3	
	Values, n^b^	Values, mean (SD)	Values, n		Values, mean (SD)	Values, n		Values, mean (SD)
Pros of drinking	56	2.34 (0.51)	N/A^c^		N/A	N/A		N/A
Cons of drinking	56	2.57 (0.50)	N/A		N/A	N/A		N/A
Information on drinking norms	55	2.67 (0.51)	N/A		N/A	N/A		N/A
Consequences of drinking	55	2.49 (0.72)	N/A		N/A	N/A		N/A
Information on BAC^d^ levels	56	2.50 (0.60)	N/A		N/A	N/A		N/A
Personal risk factors	56	2.16 (0.80)	N/A		N/A	N/A		N/A
Risks in sex or dating relationships	N/A	N/A	48		2.33 (1.06)	38		2.21 (1.02)
Ways to intervene	N/A	N/A	49		2.51 (0.89)	38		2.53 (0.76)
Consent in sexual relationships	N/A	N/A	49		2.57 (0.87)	37		2.49 (0.87)

^a^Range: 1 to 3 (1=not useful; 2=useful; 3=very useful).

^b^Reflects the number of participants completing the item.

^c^N/A: not applicable; item was not administered for this session, as content was not covered.

^d^BAC: blood alcohol content.

**Table 4:** Satisfaction with Sexual Assault and Alcohol Feedback and Education, as reported at the 6-month exit interview (n=28)^a^.

Item		Participant, n (%)
**Overall, how satisfied were you with the program?**		
	Less than moderately satisfied	1 (4)
	Moderately satisfied	5 (18)
	Very satisfied	22 (79)
**Overall, how satisfied were you with the number of sessions?**		
	Less than moderately satisfied	1 (4)
	Moderately satisfied	4 (14)
	Very satisfied	23 (82)
**Overall, how satisfied were you with the facilitators?**		
	Less than moderately satisfied	1 (4)
	Moderately satisfied	1 (4)
	Very satisfied	26 (93)
**Would you seek this program in the future for your sexual relationships?**		
	Probably not	1 (4)
	Maybe	2 (7)
	Definitely yes	25 (89)
**Would you seek this program in the future for your alcohol use?**		
	Probably not	1 (4)
	Maybe	3 (11)
	Definitely yes	24 (86)
**To what extent did this program meet your needs?**		
	Most of my needs have been met	13 (46)
	All of my needs have been met	15 (54)

^a^Sample includes only those assigned to the Sexual Assault and Alcohol Feedback and Education program.

### Primary Outcomes: Alcohol Use and Sexual Aggression

The means of the outcome variables at each time point, as well as ICCs, are displayed in Table 5. The ICCs were calculated for the 114 participants who contributed to the HLM models, given that they were the participants for whom we analyzed group differences. The results of the HLM models predicting changes in alcohol use outcomes over time are presented in Tables 6 and 7. There were no group differences in the extent to which alcohol use changed over time. Both groups showed significant declines in average number of drinks between baseline and 2 months and between baseline and 6 months. Both groups also showed significant declines in the number of heavy drinking days, but only between baseline and 6 months. Changes in the use of alcohol PBSs differed by group at 6 months, with the control group showing a decline in the use of strategies and the SAFE group showing a significant increase in the use of strategies over this time frame (effect size of standardized B=0.57).

**Table 5:** Primary outcomes at baseline, 2 months, and 6 months (n=114)^a^.

Alcohol use and sexual aggression outcomes			Baseline, mean (SD)		2 mo, mean (SD)		6 mo, mean (SD)		ICC^b^	
**Alcohol protective behavioral strategies**										0.59
	SAFE^c^	94.67 (21.73)		97.26 (32.86)		106.50 (30.08)				
	MBCC^d^	98.15 (22.06)		93.16 (24.45)		95.39 (21.14)				
**Number of heavy drinking days in the past month**										0.54
	SAFE	6.86 (4.47)		6.07 (4.53)		4.64 (4.22)				
	MBCC	6.07 (3.86)		5.43 (3.81)		4.05 (3.22)				
**Number of drinks/wk**										0.55
	SAFE	16.10 (10.22)		11.35 (10.22)		9.83 (9.57)				
	MBCC	14.91 (9.49)		11.78 (9.28)		9.75 (8.35)				
**Severity of sexual aggression**										0.18
	SAFE	6.25 (10.53)		0.85 (3.72)		2.40 (7.50)				
	MBCC	4.11 (7.19)		1.05 (4.13)		1.05 (5.31)				

^a^Values on all outcomes were not different at baseline.

^b^ICC: intraclass correlation, representing the proportion of variance due to between-person differences in the outcome.

^c^SAFE: Sexual Assault and Alcohol Feedback and Education.

^d^MBCC: mindfulness-based control condition.

**Table 6:** Group effects on alcohol protective behavioral strategy (PBS) and the number of heavy drinking days^a^.

	Alcohol PBS				Number of heavy drinking days		
	B (SE; 95% CI)	*t* test (*df*)	*P* value	B (SE; 95% CI)		*t* test (*df*)	*P* value
Intercept (baseline level)	0.03 (0.11; −0.19 to 0.25)	0.23 (112)	.82	0.11 (0.12; −0.13 to 0.35)		0.93 (112)	.36
Effect of group on baseline level	−0.14 (0.16; −0.46 to 0.18)	−0.86 (112)	.39	0.19 (0.19; −0.19 to 0.57)		1.02 (112)	.31
Change from baseline to 2-mo follow-up	−0.15 (0.11; −0.37 to 0.07)	−1.40 (112)	.17	−0.16 (0.11; −0.38 to 0.06)		−1.42 (112)	.16
Effect of group on change from baseline to 2-mo follow-up	*0.27* ^b^ *(0.16;* *−0.05 to 0.59* *)*	*1.72* *(112)*	*.09*	−0.04 (0.18; −0.40 to 0.32)		−0.19 (112)	.85
Change from baseline to 6-mo follow-up	−0.10 (0.10; −0.30 to 0.01)	−0.95 (112)	.34	−*0.46 (0.11; −0.68 to −0.24)*		−*4.20* *(112)*	*<.001*
Effect of group on change from baseline to 6-mo follow-up	*0.57 (0.18; 0.21 to 0.93)*	*3.14* *(112)*	*.002*	−0.09 (0.19; −0.47 to 0.29)		−0.50 (112)	.62

^a^Results are derived from hierarchical linear models with time (change from baseline to 2-mo follow-up and change from baseline to 6-mo follow-up) at level 1 and cross-level interactions between intervention condition at level 2 and time at level 1. Effect of group represents the difference between the intervention (coded 1) and control (coded 0) groups in the baseline levels of each outcome, amount of change between baseline and 2-month follow-up, and amount of change between baseline and 6-month follow-up. Outcome variables were standardized to interpret coefficients as effect sizes.

^b^Significant (*P*≤.05) and marginally significant (*P*≤.10) group effects are italicized. Although not presented in the table, all models included random slopes for both time components.

**Table 7:** Group effects on the number of drinks per week and severity of sexual aggression^a^.

	Drinks/wk				Severity of sexual aggression		
	B (SE; 95% CI)	*t* test (*df*)	*P* value	B (SE; 95% CI)		*t* test (*df*)	*P* value
Intercept (baseline level)	0.25 (0.13; −0.01 to 0.51)	1.93 (112)	.06	0.17 (0.13; −0.09 to 0.43)		1.35 (112)	.18
Effect of group on baseline level	0.12 (0.19; −0.26 to 0.50)	0.65 (112)	.52	0.29 (0.23; −0.17 to 0.75)		1.28 (112)	.20
Change from baseline to 2-mo follow-up	−*0.28*^b^ (0.13; *−0.54 to −0.02*)	−*2.21* *(112)*	*.03*	−0.42 (0.16; −0.74 to −0.10)		−2.69 (112)	.01
Effect of group on change from baseline to 2-mo follow-up	−0.17 (0.18; −0.53 to −0.19)	−0.98 (112)	.33	−0.33 (0.23; −0.79 to 0.13)		−1.40 (112)	.16
Change from baseline to 6-mo follow-up	−*0.48 (0.11; −0.70 to −0.26)*	−*4.198* *(112)*	*<.001*	−0.43 (0.15; −0.73 to −0.13)		−2.77 (112)	.007
Effect of group on change from baseline to 6-mo follow-up	−0.13 (0.18; −0.49 to 0.23)	−0.72 (112)	.47	−0.11 (0.26; −0.63 to 0.41)		−0.43 (112)	.67

^a^Results are derived from hierarchical linear models with time (change from baseline and 2-mo follow-up and change from baseline to 6-mo follow-up) at level 1 and cross-level interactions between intervention condition at level 2 and time at level 1. Effect of group represents the difference between the intervention (coded 1) and control (coded 0) groups in the baseline levels of each outcome, amount of change between baseline and 2-month follow-up, and amount of change between baseline and 6-month follow-up. Outcome variables were standardized to interpret coefficients as effect sizes.

^b^Significant (*P*≤.05) and marginally significant (*P*≤.10) group effects are italicized. Although not tabled, all models included significant random slopes for both time components.

### Secondary Outcomes: Peer Norms, Risks for Sexual Aggression, and Bystander Intervention

The means of the outcome variables at each time point, as well as ICCs, are displayed in Table 8. ICCs were calculated for the 114 participants who contributed to the HLM models. The results of the HLM models predicting changes in perceived peer norm outcomes over time are presented in Tables 9 and 10. Similar effects were observed for perceived peer sexual coercion and perceived peer comfort with sexism. Both declined significantly in the control group at both 2 and 6 months but declined more (to a marginally significant extent at 2 mo and significantly more at 6 mo) in the SAFE group. The effect size of this group difference in perceived peer coercion was small at 2 months (standardized B=−0.29) and nearly medium at 6 months (B=−0.47). The effect size of this group difference in peer comfort with sexism was small at 2 months (standardized B=−0.40) and medium at 6 months (B=−0.61). Peer norms for drinking declined between baseline and both 2 and 6 months *only* in the SAFE group and not in the control group. The effect size of this group difference in peer drinking norms was nearly medium at 2 months (standardized B=−0.46) and medium at 6 months (B=−0.64). Finally, a marginal group difference in the change in norms for bystander intervention was observed at 6 months only, with the SAFE group showing marginally significantly greater increases in this outcome. The effect size of this group difference was small (B=0.40).

**Table 8:** Secondary outcomes at baseline, 2 months, and 6 months (n=114)^a^.

Outcome				Baseline, mean (SD)		2 mo, mean (SD)		6 mo, mean (SD)		ICC^b^		
**Peer norms**												
	**Peer total number of drinks/wk**										0.65	
		SAFE^c^	26.82 (12.02)		21.31 (10.76)		18.91 (9.00)					
		MBCC^d^	23.66 (8.97)		22.07 (8.56)		21.91 (8.90)					
	**Peer comfort with sexism**										0.38	
		SAFE	3.71 (0.62)		3.26 (0.80)		3.11 (0.64)					
		MBCC	3.56 (0.67)		3.44 (0.87)		3.46 (0.82)					
	**Peer bystander intervention**										0.54	
		SAFE	69.02 (10.24)		72.32 (14.44)		74.27 (13.89)					
		MBCC	72.46 (10.21)		74.27 (14.45)		72.73 (13.56)					
	**Peer sexual coercion**										0.49	
		SAFE	3.49 (0.69)		2.96 (0.65)		2.75 (0.69)					
		MBCC	3.22 (0.88)		2.93 (0.79)		2.90 (0.80)					
**Risk factors for sexual aggression**												
	**Rape myth acceptance**										0.53	
		SAFE	46.52 (14.26)		42.64 (14.54)		48.19 (20.46)					
		MBCC	43.30 (12.15)		43.82 (12.69)		41.46 (14.17)					
	**Hypergender ideology**										0.65	
		SAFE	45.53 (15.92)		39.07 (13.90)		42.94 (15.78)					
		MBCC	41.51 (14.95)		42.39 (14.57)		40.99 (15.24)					
	**Labeling of consent**										0.56	
		SAFE	23.09 (4.54)		22.49 (5.96)		22.19 (5.61)					
		MBCC	23.19 (5.19)		22.92 (6.08)		23.00 (6.24)					
**Bystander intervention**												
	**Intentions to intervene**										0.49	
		SAFE	42.88 (6.73)		44.93 (6.42)		44.22 (6.87)					
		MBCC	45.27 (6.56)		42.96 (7.55)		43.82 (5.78)					
	**Confidence to help friends**										0.46	
		SAFE	853.29 (120.27)		853.05 (133.29)		859.84 (153.57)					
		MBCC	835.72 (126.18)		809.29 (174.70)		829.88 (188.98)					
	**Confidence to help strangers**										0.45	
		SAFE	415.00 (229.43)		473.33 (207.39)		540.40 (230.38)					
		MBCC	433.80 (193.23)		443.51 (226.64)		484.45 (237.08)					

^a^Values on all the outcomes were not different at baseline.

^b^ICC: intraclass correlation, representing the proportion of variance due to between-person differences in the outcome.

^c^SAFE: Sexual Assault and Alcohol Feedback and Education.

^d^MBCC: mindfulness-based control condition.

**Table 9:** Group effects on perceived norms relating to peer drinks and peer comfort with sexism^a^.

	Peer drinks				Peer comfort with sexism		
	B (SE; 95% CI)	*t* test (*df*)	*P* value	B (SE; 95% CI)		*t* test (*df*)	*P* value
Intercept (baseline level)	0.10 (0.12; −0.14 to 0.34)	0.85 (110)	.40	0.16 (0.12; −0.08 to 0.40)		1.39 (112)	.17
Effect of group on baseline level	0.31 (0.20; −0.09 to 0.71)	1.59 (110)	.12	0.20 (0.16; −0.12 to 0.52)		1.23 (112)	.22
Change from baseline to 2-mo follow-up	−0.05 (0.08; −0.21 to 0.11)	−0.64 (110)	.52	−0.18 (0.18; −0.54 to 0.18)		−1.05 (112)	.30
Effect of group on change from baseline to 2-mo follow-up	−*0.46*^b^ (0.18; *−0.82 to −0.10*)	−*2.54* *(110)*	*.01*	−*0.40 (0.21; −0.82 to 0.02)*		−*1.86* *(112)*	*.07*
Change from baseline to 6-mo follow-up	−0.12 (0.09; −0.30 to 0.06)	−1.39 (110)	.17	−0.20 (0.15; −0.50 to 0.10)		−1.32 (112)	.19
Effect of group on change from baseline to 6-mo follow-up	−*0.64 (0.17; −0.98 to −0.30)*	−*3.84* *(110)*	*<.001*	−*0.61 (0.21; −1.03 to −0.19)*		−*2.92* *(112)*	*.004*

^a^Results are derived from hierarchical linear models with time (change from baseline and 2-mo follow-up and change from baseline to 6-mo follow-up) at level 1 and cross-level interactions between intervention condition at level 2 and time at level 1. Effect of group represents the difference between the intervention (coded 1) and control (coded 0) groups in the baseline levels of each outcome, amount of change between baseline and 2-month follow-up, and amount of change between baseline and 6-month follow-up. Outcome variables were standardized to interpret coefficients as effect sizes.

^b^Significant (*P*≤.05) and marginally significant (*P*≤.10) group effects on change over time are italicized. Although not presented in the table, all models included significant random slopes for both time components.

**Table 10:** Group effects on perceived norms relating to peer bystander intervention and peer coercion^a^.

	Peer bystander intervention				Peer coercion		
	B (SE; 95% CI)	*t* test (*df*)	*P* value	B (SE; 95% CI)		*t* test (*df*)	*P* value
Intercept (baseline level)	0.03 (0.11; −0.19 to 0.25)	0.29 (110)	.77	0.19 (0.15; −0.11 to 0.49)		1.28 (112)	.20
Effect of group on baseline level	−0.29 (0.15; −0.59 to 0.01)	−1.94 (110)	.06	0.35 (0.19; −0.03 to 0.73)		1.89 (112)	.06
Change from baseline to 2-mo follow-up	0.14 (0.13; −0.12 to 0.40)	1.05 (110)	.30	−0.38 (0.11; −0.60 to −0.16)		−3.47 (112)	<.001
Effect of group on change from baseline to 2-mo follow-up	0.16 (0.19; −0.22 to 0.54)	0.86 (110)	.39	−*0.29*^b^ *(0.16; −0.61 to 0.03)*		−*1.83* *(112)*	*.07*
Change from baseline to 6-mo follow-up	0.03 (0.15; −0.27 to 0.33)	0.22 (110)	.83	−0.44 (0.14; −0.72 to −0.16)		−3.11 (112)	.002
Effect of group on change from baseline to 6-mo follow-up	*0.38 (0.20; −0.02 to 0.78)*	*1.85* *(110)*	*.07*	−*0.47 (0.20; −0.87 to −0.07)*		−*2.34* *(112)*	*.02*

^a^Results are derived from hierarchical linear models with time (change from baseline and 2-mo follow-up and change from baseline to 6-mo follow-up) at level 1 and cross-level interactions between intervention condition at level 2 and time at level 1. Effect of group represents the difference between the intervention (coded 1) and control (coded 0) groups in the baseline levels of each outcome, amount of change between baseline and 2-month follow-up, and amount of change between baseline and 6-month follow-up. Outcome variables were standardized to interpret coefficients as effect sizes.

^b^Significant (*P*≤.05) and marginally significant (*P*≤.10) group effects on change over time are italicized. Although not presented in the table, all models included random slopes for both time components.

The results of the HLM models predicting changes in sexual aggression outcomes over time are presented in Tables 11 and 12. There were no group differences in the extent to which perpetration changed over time; both groups showed significant decline in perpetration between baseline and 2 months and between baseline and 6 months. There were no group differences in rape myth acceptance. Hypergender ideology significantly declined between baseline and 2 months in the SAFE group only, and the changes at 6 months were not different by group. Finally, the labeling of sexual consent did not change in either group at either follow-up.

**Table 11:** Group effects on risk factors for sexual aggression: rape myth acceptance and hypergender ideology^a^.

	Rape myth acceptance				Hypergender ideology		
	B (SE; 95% CI)	*t* test (*df*)	*P* value	B (SE; 95% CI)		*t* test (*df*)	*P* value
Intercept (baseline level)	−0.07 (0.11; −0.29 to 0.15)	−0.68 (112)	.50	−0.04 (0.13; −0.30 to 0.22)		−0.34 (112)	.74
Effect of group on baseline level	0.22 (0.17; −0.12 to 0.56)	1.31 (112)	.19	0.27 (0.19; −0.11 to 0.64)		1.40 (112)	.16
Change from baseline to 2-mo follow-up	−0.0 (0.09; −0.23 to 0.13)	−0.49 (183)	.63	0.01 (0.13; −0.25 to 0.27)		0.04 (183)	.97
Effect of group on change from baseline to 2-mo follow-up	−0.23 (0.16; −0.55 to 0.09)	−1.44 (183)	.15	−*0.37*^b^ *(0.17; −0.71 to −0.03)*		−*2.22* *(183)*	*.03*
Change from baseline to 6-mo	−0.18 (0.10; −0.38 to 0.02)	−1.84 (183)	.07	−0.07 (0.12; −0.31 to 0.17)		−0.57 (183)	.57
Effect of group on change from baseline to 6-mo follow-up	0.28 (0.22; −0.16 to 0.72)	1.24 (183)	.22	−0.02 (0.19)		−0.08 (183; −0.40 to 0.36)	.94

^a^Results are derived from hierarchical linear models with time (change from baseline and 2-mo follow-up and change from baseline to 6-mo follow-up) at level 1 and cross-level interactions between intervention condition at level 2 and time at level 1. Effect of group represents the difference between the intervention (coded 1) and control (coded 0) groups in the baseline levels of each outcome, amount of change between baseline and 2-month follow-up, and amount of change between baseline and 6-month follow-up. Outcome variables were standardized to interpret coefficients as effect sizes.

^b^Significant (*P*≤.05) group effects on change over time are italicized. Although not presented in the table, both models included random slopes for both time components. There was a significant group difference in the change in intentions to intervene between baseline and 2 months and between baseline and 6 months (Table 12), with the control group showing a significant *decrease* in intentions to intervene between baseline and 2 months. The effect size of this group difference in intentions to intervene was medium at 2 months (standardized B=0.62) and small at 6 months (B=0.40). Confidence in the ability to help friends did not change in either group at either follow-up. There was a marginal group effect on the change in confidence in the ability to help strangers at 6 months, with the SAFE group showing a trend toward higher confidence.

**Table 12:** Group effects on risk factors for sexual aggression: labeling of sexual consent^a,b^.

	Labeling of sexual consent		
	B (SE; 95% CI)	*t* test (*df*)	*P* value
Intercept (baseline level)	0.06 (0.12; −0.18 to 0.30)	0.53 (112)	.60
Effect of group on baseline level	−0.02 (0.16; −0.34 to 0.30)	−0.12 (112)	.91
Change from baseline to 2-mo follow-up	0.01 (0.14; −0.27 to 0.29)	0.06 (184)	.95
Effect of group on change from baseline to 2-mo follow-up	−0.12 (0.19; −0.50 to 0.26)	−0.63 (184)	.53
Change from baseline to 6-mo	0.03 (0.12; −0.21 to 0.27)	0.22 (184)	.82
Effect of group on change from baseline to 6-mo follow-up	−0.19 (0.18; −0.55 to 0.17)	−1.04 (184)	.30

^a^Results are derived from hierarchical linear models with time (change from baseline and 2-mo follow-up and change from baseline to 6-mo follow-up) at level 1 and cross-level interactions between intervention condition at level 2 and time at level 1. Effect of group represents the difference between the intervention (coded 1) and control (coded 0) groups in the baseline levels of each outcome, amount of change between baseline and 2-month follow-up, and amount of change between baseline and 6-month follow-up. Outcome variables were standardized to interpret coefficients as effect sizes.

^b^Significant (*P*≤.05) group effects on change over time are italicized. For the labeling of sexual consent, random slopes were tested but were nonsignificant and removed from the model for parsimony.

There was a significant group difference in the change in intentions to intervene between baseline and 2 months and between baseline and 6 months (Tables 13 and 14), with the control group showing a significant *decrease* in intentions to intervene between baseline and 2 months. The effect size of this group difference in intentions to intervene was medium at 2 months (standardized B=0.62) and small at 6 months (B=0.40). Confidence in the ability to help friends did not change in either group at either follow-up. There was a marginal group effect on the change in confidence in the ability to help strangers at 6 months, with the SAFE group showing a trend toward higher confidence.

**Table 13:** Group effects on bystander intervention outcomes: intentions to intervene^a^.

	Intentions to intervene		
	B (SE; 95% CI)	*t* test (*df*)	*P* value
Intercept (baseline level)	0.20 (0.13; −0.06 to 0.46)	1.54 (111)	.13
Effect of group on baseline level	−0.37 (0.19; −0.75 to 0.01)	−1.99 (111)	.05
Change from baseline to 2-mo follow-up	−0.35 (0.17; −0.68 to −0.01)	−1.99 (111)	.05
Effect of group on change from baseline to 2-mo follow-up	*0.61*^b^ (0.22; 0.17 to 1.05)	*2.80 (111)*	*.01*
Change from baseline to 6-mo follow-up	−0.22 (0.15; −0.52 to 0.08)	−1.42 (68)	.16
Effect of group on change from baseline to 6-mo follow-up	*0.39 (0.19;* 0.01 to 0.77*)*	*2.01 (68)*	*.049*

^a^Results are derived from hierarchical linear models with time (change from baseline to 2-mo follow-up and change from baseline to 6-mo follow-up) at level 1 and cross-level interactions between intervention condition at level 2 and time at level 1. Effect of group represents the difference between the intervention (coded 1) and control (coded 0) groups in the baseline levels of each outcome, amount of change between baseline and 2-month follow-up, and amount of change between baseline and 6-month follow-up. Outcome variables were standardized to interpret coefficients as effect sizes.

^b^Significant (*P*≤.05) and marginally significant (*P*≤.10) group effects on change over time are italicized. Although not presented in the table, the model included a random slope for change between baseline and 2 months.

**Table 14:** Group effects on bystander intervention outcomes: confidence to help^a^.

	Confidence to help friends			Confidence to help strangers		
	B (SE)	*t* test (*df*)	*P* value	B (SE; 95% CI)	*t* test (*df*)	*P* value
Intercept (baseline level)	−0.02 (0.11; 95% CI)	−0.21 (110)	.83	−0.11 (0.12; −0.35 to 0.13)	−0.96 (110)	.34
Effect of group on baseline level	0.11 (0.16; −0.24 to 0.20)	0.67 (110)	.50	−0.10 (0.18; −0.46 to 0.26)	−0.54 (110)	.59
Change from baseline to 2-mo follow-up	−0.14 (0.17; −0.48 to 0.20)	−0.80 (110)	.42	0.05 (0.16; −0.27 to 0.37)	0.30 (110)	.76
Effect of group on change from baseline to 2-mo follow-up	0.12 (0.20; −0.28 to 0.52)	0.58 (110)	.57	0.25 (0.21; −0.17 to 0.67)	1.20 (110)	.23
Change from baseline to 6-mo follow-up	−0.11 (0.21; −0.53 to 0.31)	−0.52 (110)	.60	0.16 (0.18; −0.20 to 0.52)	0.89 (110)	.38
Effect of group on change from baseline to 6-mo follow-up	0.16 (0.24; −0.32 to 0.64)	0.68 (110)	.50	*0.43*^b^ (0.23; *−0.03 to 0.89*)	*1.86* *(110)*	*.07*

^a^Results are derived from hierarchical linear models with time (change from baseline to 2-mo follow-up and change from baseline to 6-mo follow-up) at level 1 and cross-level interactions between intervention condition at level 2 and time at level 1. Effect of group represents the difference between the intervention (coded 1) and control (coded 0) groups in the baseline levels of each outcome, amount of change between baseline and 2-month follow-up, and amount of change between baseline and 6-month follow-up. Outcome variables were standardized to interpret coefficients as effect sizes.

^b^Significant (*P*≤.05) and marginally significant (*P*≤.10) group effects on change over time are italicized. Although not presented in the table, all models included random slopes for both time components.

### Proximal Session 1 Outcomes

Repeated measures analyses of variance that examined changes from before session to immediately after session revealed that from baseline to after session 1, the men in SAFE reported greater increases in the motivation to change their alcohol use than the men in MBCC. The groups were not significantly different in the extent to which the showed an increase in self-efficacy or a decrease in weekly drinking intentions (Table 15).

**Table 15:** Proximal session 1 outcomes: motivation to change, self-efficacy, and intentions to drink^a^.

Outcome		Baseline, mean (SE)	Session 1 posttest, mean (SE)	*F* test (*df*)		*P* value	
**Self-efficacy in changing drinking (n=104)**					1.49 (1,102)		.23
	SAFE^b^	74.53 (4.10)	79.40 (3.72)				
	MBCC^c^	79.39 (4.35)	80.71 (3.94)				
**Motivation to change drinking (n=110)**					4.52 (1,108)		.04
	SAFE	2.46 (0.41)	3.87 (0.44)				
	MBCC	2.91 (0.41)	3.22 (0.44)				
**Intended total number of drinks/wk (n=109)**					2.92 (1,107)		.09
	SAFE	21.87 (2.20)	14.43 (1.14)				
	MBCC	19.55 (2.18)	16.84 (1.13)				

^a^Results are based on repeated measures analyses of variance. Responses are shown from all men who completed session 1. There were no significant group differences at baseline.

^b^SAFE: Sexual Assault and Alcohol Feedback and Education.

^c^MBCC: mindfulness-based control condition.

## Discussion

### Primary Findings

This randomized pilot trial of an integrated alcohol and sexual assault prevention program, SAFE, advances the science and practice of sexual assault prevention by documenting the feasibility, acceptability, and preliminary outcomes associated with the program. The SAFE program is a 3-session integrated alcohol and sexual assault intervention for college men who engage in heavy drinking grounded in social norms theory and bystander intervention. The program is set apart from other sexual assault prevention programs through its integration of well-established alcohol intervention strategies (ie, BMI+PNF) into the curriculum. The evaluation is also unique in the use of an active mindfulness-based comparison condition, which was well matched to the dose and delivery method of the SAFE program.

Completion rates were >80% for each session of the SAFE program, as well as the MBCC program, suggesting that the programs were feasible to implement (research question 1). Over 80% of each group completed assessments at both 2 months and 6 months, suggesting that the research procedures were successful in retaining participants and preventing differential dropout between groups (research question 2). A review of the audio recordings of each SAFE session and MBCC session indicated that the facilitators were adequately trained in administering the sessions according to the manual (84%-99% of the content implemented on average; research question 3), with high levels of competency in the intervention delivery study (research question 4). Ratings of satisfaction and program utility were also high (research question 5). Given that sexual assault prevention programs are often perceived to incite defensiveness among college men [39], the high receptivity to this program is a significant positive outcome. Although speculative, the use of an interactive, discussion-oriented MI style, which emphasizes participant choice, may help men engage in sexual assault prevention in a nondefensive manner.

Although the focus of a pilot study is to demonstrate the acceptance of a new intervention, ensure the ability to recruit and maintain participants in a research protocol, and establish the feasibility of intervention delivery [50], a series of exploratory outcomes were administered to explore potential changes in the putative program effects. Measures were aligned with the logic model of the program and would be planned for use in a larger randomized clinical trial. Some promising findings emerged.

Regarding the primary outcomes of alcohol use and sexual aggression, although the extent of alcohol use did not vary by group over time, both groups reported lower alcohol use over the course of the study. Specifically, both groups showed significant declines in average number of drinks between baseline and 2 months and between baseline and 6 months. Both SAFE and MBCC participants also showed significant declines in the number of heavy drinking days between baseline and 6 months. These results are positive and not surprising given prior research suggesting that BMI+PFR and alcohol interventions that include relaxation training demonstrate positive outcomes for drinking quantity and frequency [43,44]. Alcohol PBSs varied by group following program participation such that the participants in the SAFE intervention reported increases in PBSs from baseline to 6 months compared with the participants in the MBCC group, who showed decreases in PBSs from baseline to 6 months. This finding is notable, given that prior studies suggest that PBSs mediate changes in alcohol use following motivational interventions [80]. Although speculative, the SAFE program gave considerable attention to developing an individual change plan focused on alcohol PBSs and provided participants with a tip sheet listing various alcohol PBSs that they could implement. These program components may have supported the development of alcohol PBSs among the SAFE participants. It is unclear why the MBCC participants showed decreases in PBSs over time, and additional research is required to better understand this effect.

The severity of sexual aggression did not vary between the SAFE and MBCC groups at 2 or 6 months. Changes in sexual aggression severity over time for each group were not examined, given that this outcome was assessed with respect to varying periods at each assessment (ie, since the age of 14 years at baseline, past 2 mo at the 2-mo follow-up, and past 4 mo at the 6-mo follow-up). However, regarding the secondary outcomes of peer norms, risk factors for sexual aggression, and bystander intervention, several positive findings were evidenced. Specifically, when examining the impacts of perceived peer norms, similar effects were observed for perceived peer sexual coercion and perceived peer comfort with sexism such that both outcomes declined significantly in the control group at 2 and 6 months but declined more in the SAFE group over time. The SAFE intervention is grounded in Berkowitz’s Integrated Model of Sexual Assault [39], which theorizes that perceived peer norms interact with other individual characteristics and environmental factors to increase perpetration proclivity. These results support this theory. In addition, only the participants in the SAFE group reported declines in perceived peer norms for drinking between baseline and 2 months and between baseline and 6 months. At 6 months, only the SAFE group showed marginally significant increases in the perceptions of peer engagement in bystander intervention.

Although these findings should be interpreted cautiously given the small sample size in this study, they suggest that the SAFE intention has positive impacts on several of the perceived norms that are targeted in the program, supporting previous research on the importance of incorporating misperception correction and bystander intervention skills training in sexual assault prevention programs for men [39]. Experimental studies suggest that when men’s misperception of peer engagement in aggressive behavior is reduced, their proclivity for personal engagement in violence is also reduced [81].

Regarding risk factors for sexual aggression, SAFE showed a decline in the endorsement of hypergender ideology at 2 months compared with baseline. These findings are positive given that numerous studies document a link between ascription to traditional masculine norms and perpetration of sexual aggression among men [82]. Neither group showed a decline in rape myth acceptance or the labeling of sexual consent. Although the findings specific to rape myth acceptance are surprising, more recent findings suggest that the effect of adherence to rape myths on sexual aggression perpetration may be moderated by levels of perceived peer approval, which may prove to be a more proximal intervention target for prevention programs [83]. Furthermore, although it is also unexpected that men’s ability to label sexual consent within a written vignette did not change over time, it is plausible that men’s ability to recognize more nuanced aspects of sexual consent in real-life situations, including when alcohol is involved, did change following the intervention.

Given that not all men who engage in heavy drinking have a history of perpetration or might be at risk of engaging in perpetration in the future, the SAFE program focused on engaging all men as active bystanders in addressing situations that pose a risk for violence in their campus community. When examining program impacts on bystander intervention outcomes, it was surprising that the control group showed a significantly decrease in intentions to intervene between baseline and 2 months. Qualitative inquiry is warranted to better understand why the men in the MBCC group showed a decrease in this outcome. In addition to examining intentions to intervene and self-efficacy in helping, future studies should include measures of actual engagement in bystander intervention. SAFE provided extensive skills-based practice in bystander intervention strategies, which may explain the marginal group effect on change in confidence in the ability to help strangers at 6 months, with the SAFE participants showing a trend toward higher confidence. Further exploration is warranted to determine why confidence in the ability to help friends did not change for either group at any reporting period.

Proximal outcomes related to the proposed mechanisms of change in session 1 were also examined. The men in the SAFE group reported higher levels of motivation to change alcohol use following session 1 than the men in the MBCC group. The participants in the SAFE group did not report changes in self-efficacy or weekly drinking intentions when assessed after session relative to the participants in the MBCC group. However, both groups were moving in the expected positive direction for each of these outcomes (ie, increased self-efficacy and lower drinking intentions).

Although this study supports the feasibility, acceptability, and utility of the SAFE program, several considerations should be noted when interpreting the findings. Statistical evaluation of program outcomes should be interpreted with caution, given the tendency of small-scale pilots to result in type II errors [51,52]. In addition, the study limited recruitment to a single university in the Northeastern United States. Aligning with the demographics of the university, the racial and ethnic diversity in the sample was limited. It should also be noted that this study did not assess sex assigned at birth or gender identity. Rather, potential participants were invited to the research study if they were listed as self-reporting their gender identity as a “man” on the university registrar record. Given that the sexual assault prevention program addressed men’s perpetration of sexual aggression against women, participants were included in the study if they had engaged in prior sexual activity with a female partner. Sexual orientation was not assessed in this study. It is possible that the intervention would be useful for men regardless of their engagement in recent sexual activity and for men who are sexually active with men, but this is not known. Future work is warranted to also ensure that programs address the prevention of sexual aggression toward any individual, regardless of gender.

The study also implemented a restricted set of inclusion criteria relating to alcohol use, with the goal of enrolling a high-risk sample, and participants were restricted to men who had engaged in recent binge drinking. The intervention may also be useful for men who consume alcohol but do not engage in binge drinking. Research is also warranted to understand ideal ways to target men who engage in binge drinking or other forms of problematic alcohol use (eg, hazardous drinking) in sexual assault prevention efforts. Given the likelihood for men with higher levels of aggressive behavior to respond differently to the intervention, future analyses in larger samples should explore the possibility for intervention effects to vary as a function of men’s baseline history of perpetration.

### Conclusions

In summary, the SAFE intervention integrates social norms theory, bystander intervention, and a nonjudgmental MI approach to target both alcohol use and sexual aggression in high-risk college men. This study documents the potential for the SAFE program to serve as a targeted sexual assault prevention program that addresses both sexual aggression proclivity and alcohol use outcomes among college men who engage in heavy drinking. The development of sexual assault prevention programs specifically targeting high-risk groups is lacking [29], and further work is needed to continue to develop interventions that target the role of alcohol use in sexual assault.

## Appendix

Multimedia Appendix 1 CONSORT (Consolidated Standards of Reporting Trails) checklist.

## Abbreviations

ASPD: antisocial personality disorder
CSQ-8: Client Satisfaction Questionnaire-8
HLM: hierarchical linear modeling
ICC: intraclass correlation
MBCC: mindfulness-based control condition
MI: motivational interviewing
PBS: protective behavioral strategy
PNF: personalized normative feedback
SAFE: Sexual Assault and Alcohol Feedback and Education

## Data Availability

Data are available upon request by contacting the primary author.
